# Methyl lucidone induces apoptosis and G_2_/M phase arrest *via* the PI3K/Akt/NF-κB pathway in ovarian cancer cells

**DOI:** 10.1080/13880209.2019.1701044

**Published:** 2019-12-25

**Authors:** Jae-Hwan Yoon, Jong-Woon Shin, Thu-Huyen Pham, Youn-Jin Choi, Hyung-Won Ryu, Sei-Ryang Oh, Jae-Wook Oh, Do-Young Yoon

**Affiliations:** a Department of Bioscience and Biotechnology, Research Institute of Bioactive-Metabolome Network, Konkuk University, Seoul, Republic of Korea; b Department of Obstetrics and Gynecology, Seoul St. Mary’s Hospital, College of Medicine, The Catholic University of Korea, Seoul, Republic of Korea; c Natural Medicine Research Center, Korea Research Institute of Bioscience and Biotechnology, Chungcheungbuk-do, Republic of Korea; d Department of Stem Cell and Regenerative Biotechnology, Konkuk University, Seoul, Republic of Korea

**Keywords:** *Lindera erythrocarpa* Makino, cell death, cell cycle arrest, OVCAR-8, SKOV-3

## Abstract

**Context:**

Methyl lucidone (ML) from the dried fruit of *Lindera erythrocarpa* Makino (Lauraceae) exhibits cytotoxic effects in various cancer cell lines. However, its effects on ovarian cancer cells remain unknown.

**Objective:**

This study evaluates the mechanism of ML-induced apoptosis, cell cycle distribution in ovarian cells.

**Materials and methods:**

The cytotoxic effect of ML (2.5–80 µM) on OVCAR-8 and SKOV-3 cells was evaluated by MTS assay for 24 and 48 h. Apoptosis and cell cycle arrest were analysed by flow cytometry. PCR, western blot analyses were performed to examine the related signalling pathways.

**Results:**

ML induced significant cellular morphological changes and apoptosis in ovarian cancer cells, leading to an antiproliferative effect (IC_50_ = 33.3–54.7 µM for OVCAR-8 and 48.8-60.7 µM for SKOV-3 cells). Treatment with ML induced cleavage of caspase-3/9 and PARP and release of cytochrome c from the mitochondria. Moreover, ML downregulated the expression of Bcl-2 and Bcl-xL and induced cell cycle arrest in the G_2_/M phase. Additionally, ML suppressed the expression of cyclin-A/B and promoted that of the cyclin-dependent kinase inhibitors p21 and p27. The expression of death receptors was not altered. Interestingly, ML also inhibited the activity of PI3K/Akt and NF-κB.

**Discussion and conclusions:**

ML caused G_2_/M phase arrest and apoptosis in ovarian cancer cells by activating intrinsic apoptotic pathways and suppressing the PI3K/Akt survival pathway. ML may be a potential anticancer agent to suppress ovarian cancer proliferation; thus, to improve the survival rate of cancer patients.

## Introduction

Ovarian cancer is the fifth most common cause of gynaecological cancer-related mortality in the United States of America, with an estimated 14,000 deaths recorded in 2017 (Siegel et al. 2017). In 2018, approximately 295,000 cases and 180,000 deaths were reported worldwide (Bray et al. 2018). Early detection of ovarian cancer is difficult owing to the lack of symptoms, which leads to a low survival rate (less than 30%) or progression to peritoneal metastasis (Ye et al. 2014). Therefore, there is an urgent need for novel chemotherapeutic agents to improve the survival rate of these patients.

Apoptosis, or programmed cell death, consists of biochemical events that lead to morphological changes, including membrane blebbing and cell shrinkage (Kerr et al. 1972). Apoptosis is initiated by two distinct pathways: the intrinsic and the extrinsic pathways (de Bruin and Medema 2008). The intrinsic pathway is triggered by growth factors and oxidative stress and is dependent on the mitochondria, whereas the extrinsic pathway is induced by cell surface receptors. Especially, dissipation of the mitochondrial membrane potential (MMP) triggers apoptosis by releasing apoptotic proteins (Petros et al. 2004; de Bruin and Medema 2008). These two pathways eventually converge through the caspase cascades (Li et al. 1997). Various cancer types elude these apoptotic pathways, promoting cancer survival and resistance to chemotherapeutic agents. Therefore, controlling pivotal apoptosis regulators is an effective strategy in cancer therapy (Lu et al. 2008; Xu et al. 2017).

Methyl lucidone (ML) is isolated from the dried fruit of *Lindera erythrocarpa* Makino (Lauraceae). This plant, distributed throughout China, Japan and Korea, is a traditional medicine known for its antifungal, digestive and antibacterial activities. Studies have reported antiinflammatory and neuroprotective effects of ML (Cui et al. 2012; Wang et al. 2008). Jin et al. (2018) reported that ML inhibits STAT3 activity *via* suppression of MEG2 in prostate cancer cells. However, the mechanisms underlying the apoptotic effects of ML in ovarian cancer remain unknown; thus, this effect was evaluated to assess the potential of ML as a chemotherapeutic agent. Here, we demonstrated for the first time, to our knowledge, that ML induces apoptosis by suppressing the PI3K/Akt survival pathway and activating the intrinsic apoptotic pathway in OVCAR-8 and SKOV-3 ovarian cancer cells.

## Materials and methods

### Preparation of ML

*Lindera erythrocarpa* fruit was newly collected from Jeju Island, Korea, in October 2013, and identified by Dr. Jin Hyub Paik at Korea Research Institute of Bioscience & Biotechnology (KRIBB, Ohchang, Republic of Korea). A voucher specimen (KRIB 0000372) was deposited in the Herbarium of the KRIBB (Ohchang, Republic of Korea). The dried fruits (5.0 kg) were extracted with methanol (15 L × 3) at room temperature (RT) to obtain about 770.0 g of solid extract, which was then fractionated on a silica gel column (10 × 90 cm, JEO prep 60, 40–63 μm, 2.3 kg, Zeochem, Louisville, KY) and eluted using hexane-EtOAc mixtures (20:1→15:1→10:1→8:1→6:1→4:1→2:1→1:1) to give 10 pooled fractions (LE Frs. 1–10), which were combined based on a comparison of their thin layer chromatography (TLC) and ultra-performance liquid chromatography (UPLC)-photodiode array detection (PDA) profiles. LE Fr. 8 (35.4 g) was purified by medium pressure liquid chromatography (MPLC) (Spot Prep II 250, Armen, Paris, France, flow rate: 100 mL/min) using a YMC ODS AQ HG (10 × 250 mm, 10 μm, Kyoto, Japan) and a gradient solvent system (0–50.0 min, 60% MeOH; 50.0–70.0min, 60–100% MeOH) to yield ML (2.4 g). Finally, the purified ML was identified by comparing its nuclear magnetic resonance (NMR), ultraviolet–visible spectrophotometry (UV), mass spectrometry (MS), MS-MS and high resolution mass spectrometry (HRMS) spectral data with published date from literature (Lee et al. 2015). Yellow solid; UV (MeOH) *λ*_max_ nm 240, 352; ^1^H NMR (400 MHz, CDCl_3_) *δ* 7.97 (1H, d, *J*= 15.6 Hz, H-α), 7.60 (1H, d, *J*= 15.9 Hz, H-β), 7.58 (1H, m, H-2, 6), 7.36 (1H, m, H-3, 4, 5), 5.92 (1H, s, H-4′), 4.17 (3H, s, β′-OCH_3_), 3.91 (3H, s, 3′-OCH_3_); ^13^C NMR (100 MHz, CDCl_3_) 191.8 (C-5′), 185.6 (C-2′), 170.2 (C-3′), 169.0 (C-β′), 142.9 (C-β), 135.6 (C-1), 130.5 (C-4), 129.1 (C-3, 5), 128.7 (C-2, 6), 121.7 (C-α), 111.9 (C-4′), 109.4 (C-1′), 64.8 (C-β′-OCH_3_), 58.7 (C-3′-OCH_3_); HRESIMS *m/z* [M + H]^+^ 271.0983 (calculated for C_16_H_15_O_4_, 271.0970).

### Reagents

The stock solution of ML (100 mM) in dimethyl sulphoxide (DMSO) was stored in the dark at 4 °C and diluted in Roswell Park Memorial Institute (RPMI)-1640 medium immediately before use (Hyclone, Rockford, IL). CellTiter 96 AQueous One Solution Cell Proliferation Assay reagent [3-(4,5-dimethylthiazol-2-yl)-5-(3-carboxymethoxyphenyl)-2-(4-sulfophenyl)-2H-tetrazolium (MTS)] and propidium iodide (PI) were purchased from Promega (Madison, WI) and Millipore Sigma (Burlington, MA), respectively. NE-PER Nuclear and Cytoplasmic Extraction reagents were obtained from Thermo Fisher Scientific Inc (Waltham, MA). Mitochondria/Cytosol Fraction kit reagents were purchased from Biovision (Milpitas, CA). The RIPA buffer was obtained from DyneBio (Seoul, Republic of Korea). The general caspase inhibitor Z-VAD-FMK was supplied by R&D systems (Minneapolis, MN). Doxorubicin was obtained from Millipore Sigma (Burlington, MA). Antibodies specific to procaspase-3/cleaved caspase-3, procaspase-9/cleaved caspase-9, PARP, β-actin, Bcl-xL, Bcl-2, Bax, IκBα, p-IκBα and p-Akt1/2/3 (Ser473) were purchased from Cell Signalling Technology (Beverly, MA). The anti-rabbit IgG horseradish-peroxidase (HRP)-conjugated secondary antibody and the anti-mouse IgG HRP conjugated secondary antibody were purchased from Millipore Sigma (Burlington, MA). Antibodies specific to cyclin D1, cyclin A, cyclin B, cyclin E, p21, p27, PI3K, Akt and NF-κB were purchased from Santa Cruz Biotechnology (Dallas, TX). Antibodies specific to cytochrome c and lamin B1 were purchased from Novus Biologicals (Centennial, CO) and GeneTex (Irvine, CA), respectively. JC-1 (5,50,6,60-tetrachloro-1,10,3,30-tetraethyl benzimidazoly-carbocyanine chloride) was obtained from Enzo (Farmingdale, NY). The fluorescein isothiocyanate (FITC)-Annexin V Apoptosis Detection kit was supplied by BD Biosciences (San Jose, CA). 1D (1H, 13C and DEPT) NMR spectra were obtained on Bruker AM 400 spectrometers (Bruker, Madison, WI) with tetramethylsilane (TMS) as the internal standard. High- resolution electrospray ionization mass spectrometry (HRESIMS) was performed on a Waters ACQUITY UPLC system (Waters Corporation, Milford, MA), equipped with a binary solvent delivery system, autosampler and UV detector combined with a Micromass Q-Tof Premier™ mass spectrometer (Waters Corporation, Milford, MA) system in the positive-ion mode.

### Cell lines

The human ovarian adenocarcinoma cell line, OVCAR-8, was obtained from the National Cancer Institute (Rockville, MD) and SKOV-3 was obtained from the American Type Culture Collection (Manassas, VA). Cells were cultured in RPMI medium containing 10% (v/v) heat-inactivated foetal bovine serum (Millipore Sigma, Burlington, MA) at 37 °C in an atmosphere of 5% CO_2_.

### Cell viability assays

Cell viability was assessed using MTS assay, according to the manufacturer’s instructions. Cells (1 × 10^4^ cells/mL) were seeded in 96-well plates and treated with various concentrations of ML for 24or 48 h. Optical absorbance was measured at 492 nm using an enzyme-linked immunosorbent assay reader (Apollo LB 9110, Berthold Technologies, Bad Wildbad, Germany).

### Flow cytometry analysis

Cells (1.5 × 10^5^ cells/mL) were treated with ML for 24 h, and then harvested and washed with phosphate-buffered saline (PBS) before exposure to staining buffer. Annexin V and PI staining were done using the FITC-Annexin V Apoptosis Detection kit, according to the manufacturer’s instructions. For cell cycle analysis, harvested cells were fixed with 80% ethanol, washed twice with cold PBS and centrifuged to discard the supernatant. The pellet was resuspended in PI staining buffer, containing 50 μg/mL PI and 100 μg/mL RNase A in PBS, for 20 min in the dark. JC-1 staining was used to analyse MMP. Cells were stained with JC-1 staining buffer (5 μg/mL) and incubated in the dark for 10 min, washed twice with PBS and resuspended in 200 μL PBS. The data were analysed using a FACSCalibur instrument and Cell Quest software (BD Biosciences, San Jose, CA).

### Western blot analysis

Cells were treated with the indicated ML concentrations for 24 h. They were harvested and lysed in RIPA buffer containing a protease inhibitor cocktail (Roche Diagnostics, Mannheim, Germany). The nuclear and cytoplasmic parts were fractionated using the NE-PER, whereas the mitochondrial and cytosol parts were fractionated using Mitochondria/Cytosol Fraction kit according to the manufacturer’s instructions. The protein content was quantified using the Bradford assay (BioRad, Hercules, CA). The cell lysate was separated by 10–15% SDS polyacrylamide gel electrophoresis. The proteins were transferred onto polyvinylidene difluoride membranes (Millipore Sigma, Burlington, MA), which were blocked in 5% powdered skim milk dissolved in Tris-buffered saline containing 0.1% Tween-20 for 1 h at RT. The membranes were incubated overnight at 4 °C with specific primary antibodies. After washing, the membranes were incubated with the secondary antibodies for 1 h at RT. The blots were detected using a chemiluminescence detection kit (Advanstar, Cleveland, OH). The relative intensity of the western blot bands was measured using ImageJ version 1.52a software (NIH, Bethesda, MD).

### RT-PCR

Cells treated with ML were harvested; their RNA was extracted using an easy-BLUE^™^ total RNA extraction kit (iNtRON Biotechnology, Seongnam, Republic of Korea), according to the manufacturer’s instructions. cDNA products were obtained using M-MuLV reverse transcriptase (New England Biolabs, Ipswich, MA). Each sample contained one of the following primer sets: *DR5*, 5′-CACCTTGTACACGATGCTGA-3′ (forward), 5′-GCTCAACAAGTGGTCCTCAA-3′ (reverse); *DR6*, 5′-TGCAGTATCCGGAAAAGCTC-3′ (forward), 5′-TCTGGGTTGGAGTCATGGAT-3′ (reverse); *FADD*, 5′-GGGGAAAGATTGGAGAAGGC-3′ (forward), 5′-CAGATTCTCAGTGACTCCCG-3′ (reverse); *TRADD*, 5′-CTATTGCTGAACCCCTGTCC-3′ (forward), 5′-AGAATCCCCAATGATGCACC-3′ (reverse); *FAS*, 5′-AGGGATTGGAATTGAGGAAG -3′ (forward), 5′-ATGGGCTTTGTCTGTGTACT-3′ (reverse); *β-Actin*, 5′-TCATGAAGTGTGACGTGGAC-3′ (forward), 5′-GCAGTGATCTCCTTCTGCAT-3′ (reverse).

### Statistical analysis

The data are presented as means ± SEM (*n* = 3). One-way analysis of variance (ANOVA), followed by Tukey’s HSD test, was used to determine differences between groups. *p < 0.05 or **p < 0.01 were considered statistically significant.

## Results

### ML inhibits the viability of OVCAR-8 and SKOV-3 ovarian cancer cells in a dose-dependent manner

**Figure 1.**
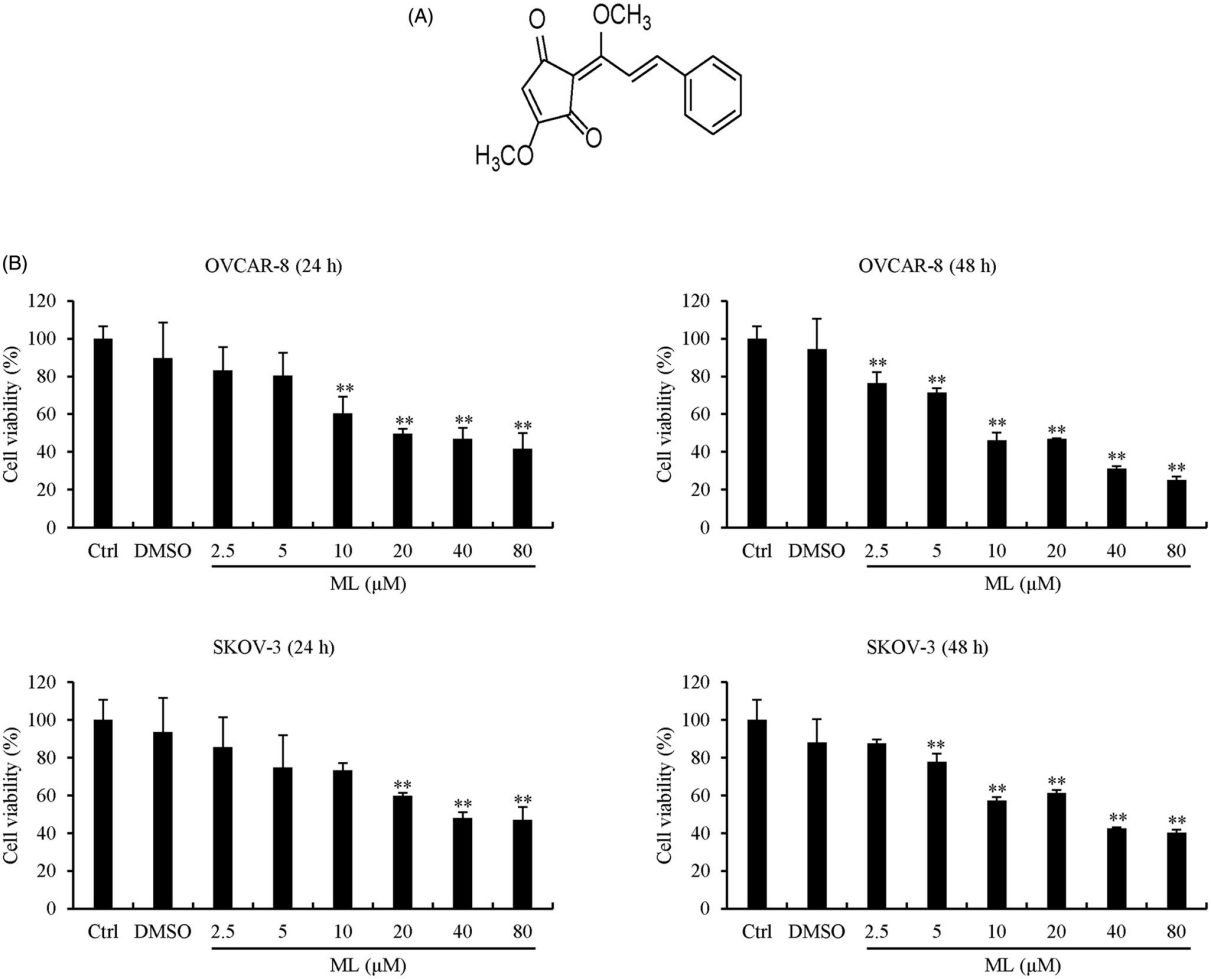
Structure of ML and the effect of ML on cell viability. (A) Structure of ML, a cyclo-pentenedione isolated from the dried fruit of *L. erythrocarpa*. (B) OVCAR-8 and SKOV-3 cells were treated for 24 and 48 h with the indicated amounts of ML; cell viability was assessed by the MTS assay. Results represent the mean ± SEM of three experiments (significant *vs.* control, *p< 0.05, **p< 0.01).

The *in vitro* antitumor effect of ML on two ovarian cell lines was assessed using the MTS assay. OVCAR-8 and SKOV-3 cells were treated with concentrations of ML up to 80 μM for 24 and 48 h. As shown in Figure 1(B), cell viability was reduced in a time and dose-dependent manner and was significant compared to untreated cells (*p<* 0.05). IC_50_ values were approximately 54.716 and 60.708 µM at 24 h and 33.317 and 48.827 µM at 48 h on OVCAR-8 and SKOV-3, respectively.

### ML induces apoptosis and morphological changes

**Figure 2.**
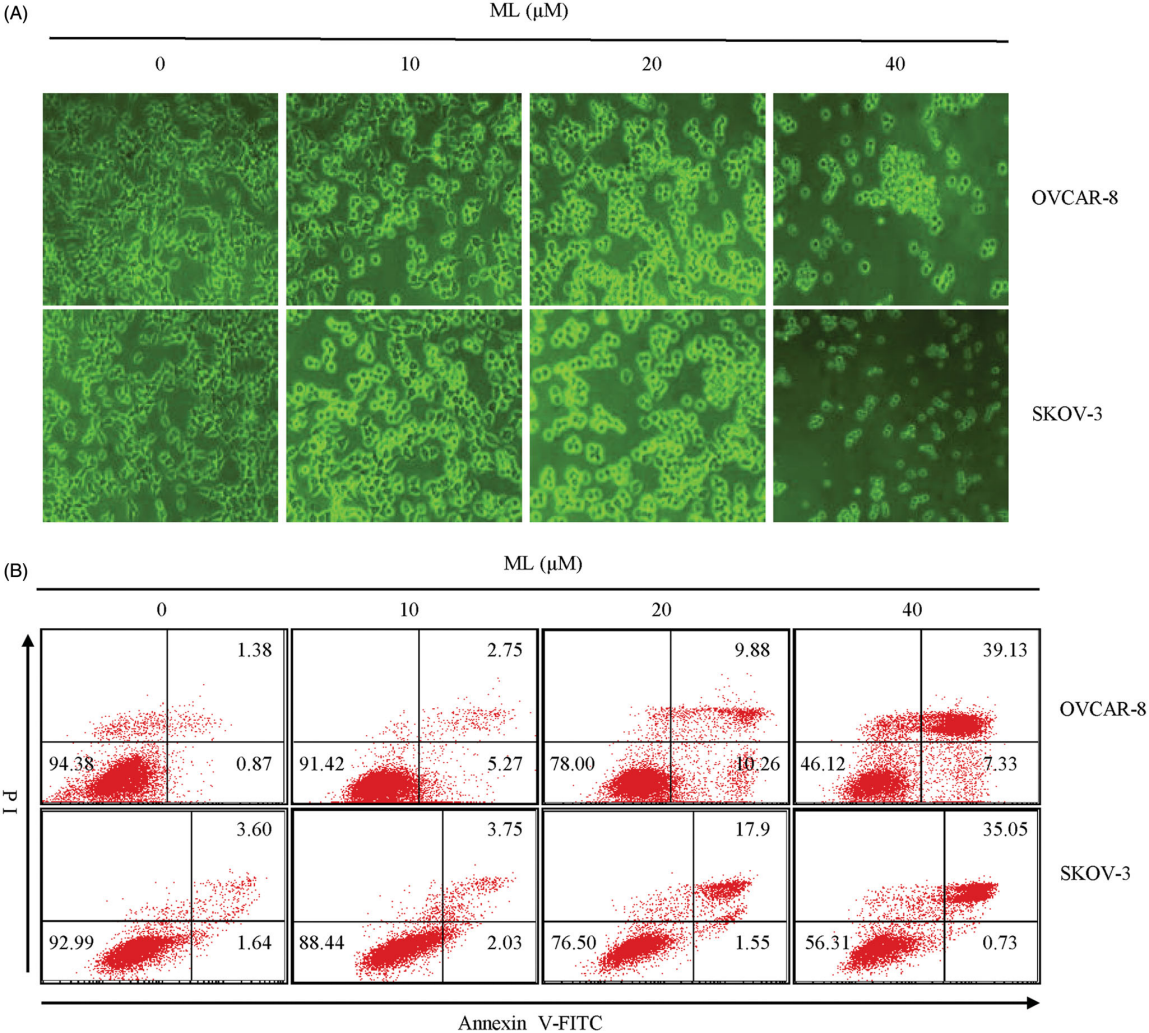
Effects of ML on apoptosis. OVCAR-8 and SKOV-3 cells were treated with the indicated amounts of ML for 24 h. (A) Cells under a phase-contrast microscope at 100× magnification. (B) ML-pre-treated cells were stained with annexin V-FITC/PI and analysed by flow cytometry. The lower left quadrant shows viable cells, the lower right quadrant shows early apoptotic cells and the upper right quadrant shows late apoptotic cells.

We investigated whether ML could induce morphological changes, such as cellular shrinkage and membrane blebbing, which are typical characteristics of apoptosis. Phase-contrast microscopy revealed that cells treated with various concentrations of ML for 24 h exhibited morphological changes culminating in cell death (Figure 2(A)). Flow cytometry after FITC-annexin V/PI staining was used to determine whether ML caused cellular apoptosis. Compared to untreated cells, ML induced apoptosis in both cell lines in a dose-dependent manner. The apoptotic rates of OVCAR-8 cells were 2.25%, 8.02%, 20.14% and 46.46% at concentrations of 0, 10, 20, 40 µM of ML, respectively; regarding SKOV-3 cells, these values were 5.24%, 5.78%, 19.45% and 35.78% at the same ML concentrations (Figure 2(B)). The results showed a shift of the ML-treated cells from the normal to the apoptotic phase.

### ML activates apoptosis via the intrinsic pathway

**Figure 3.**
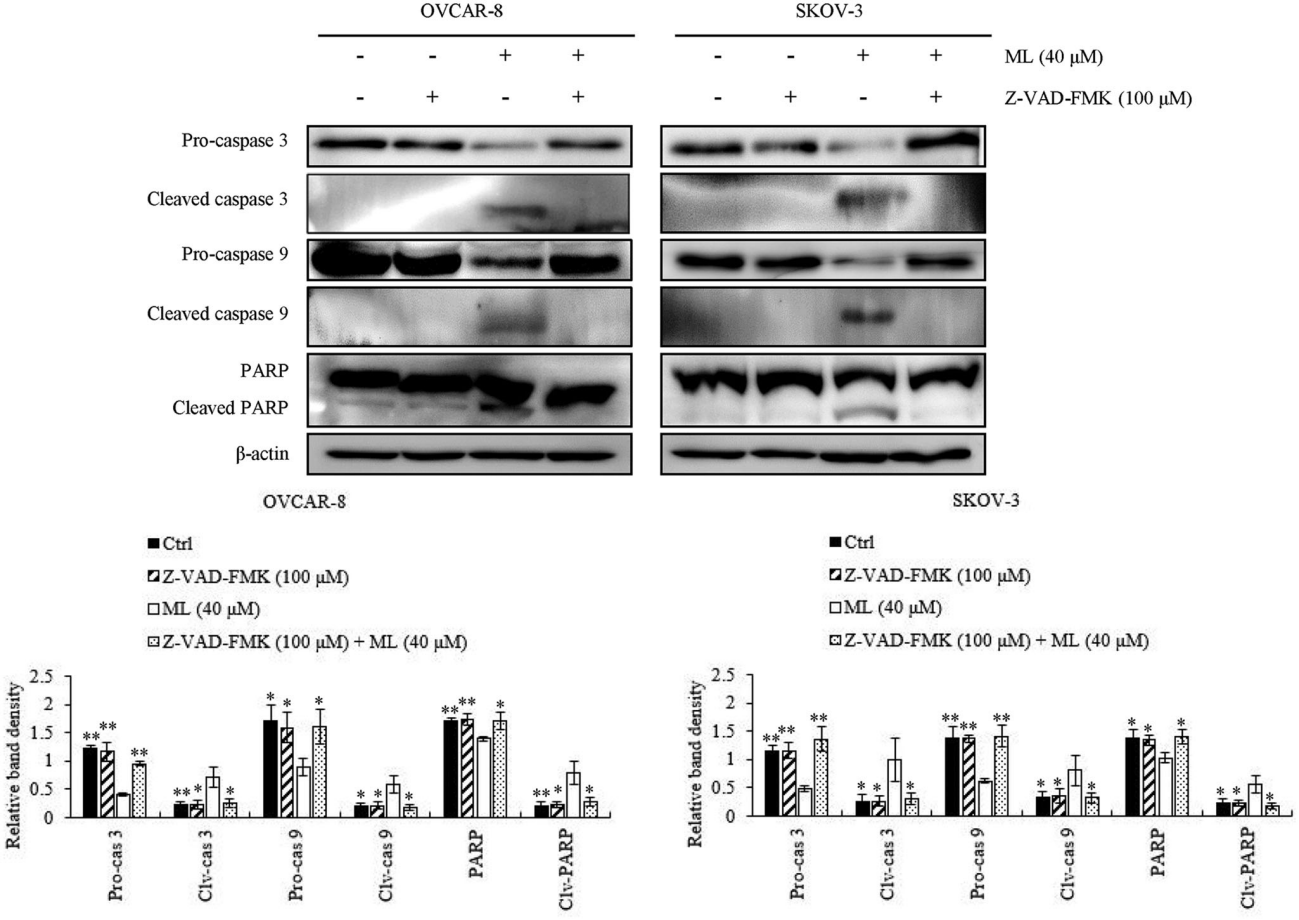
Effect of the pan-caspase inhibitor Z-VAD-FMK on ML-pre-treated cells. OVCAR-8 and SKOV-3 cells were pre-treated with Z-VAD-FMK (100 μM) for 5 h and with ML (40 μM) for 24 h. Western blotting analyses of the procaspase-3 (Pro-cas 3), cleaved caspase-3 (Clv-cas 3), procaspase-9 (Pro-cas 9), cleaved caspase-9 (Clv-cas 9), PARP and cleaved PARP (Clv-PARP). Western blotting used whole-cell lysates and specific antibodies for the pro-/cleaved-form of caspase-9, caspase-3 and PARP. Results represent the mean ± SEM of three experiments (significant *vs*. ML-treated cells, *p< 0.05, **p< 0.01).

**Figure 4.**
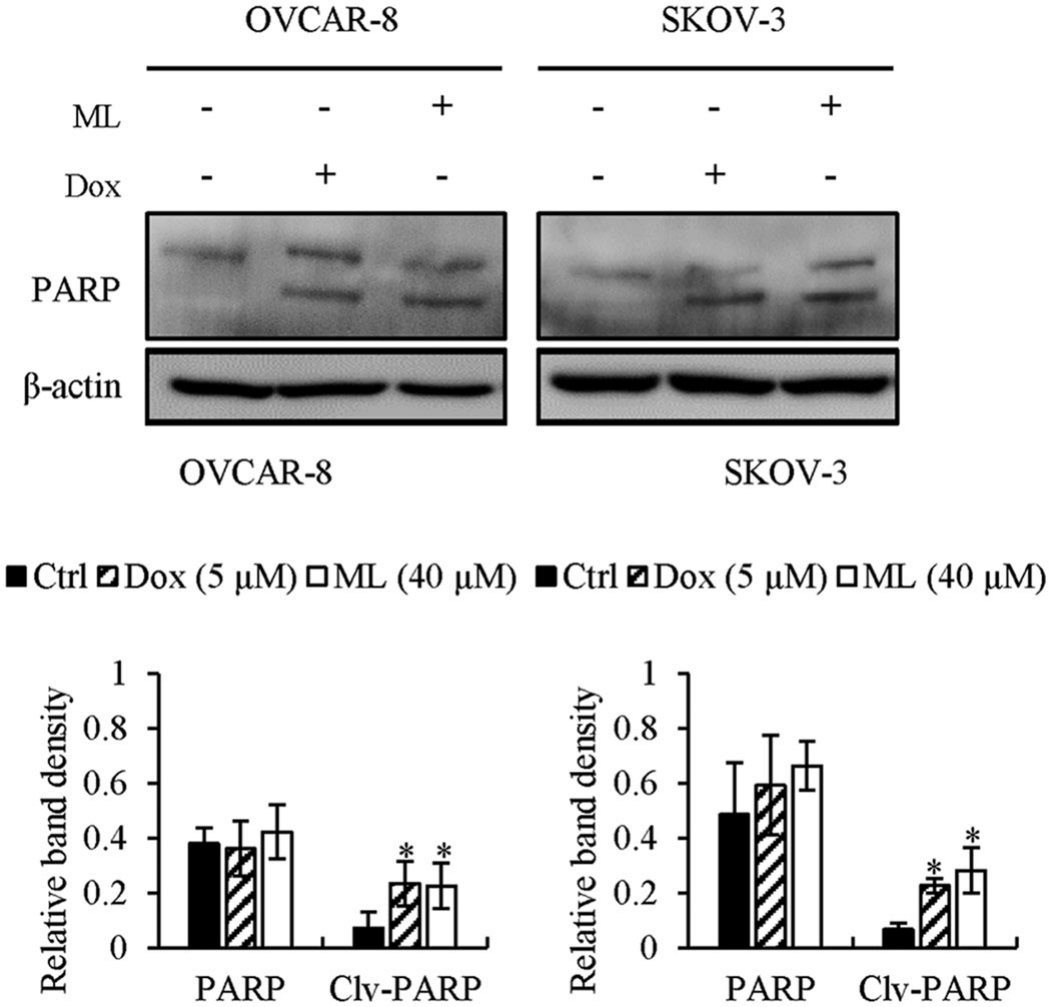
The effect of ML or doxorubicin on PARP cleavage. Cells were treated with doxorubicin (5 µM) or ML (40 µM) and then, incubated for 24 h. PARP cleavage was detected by western blotting. Results represent the mean ± SEM of three experiments (significant *vs.* control, *p< 0.05, **p< 0.01).

**Figure 5.**
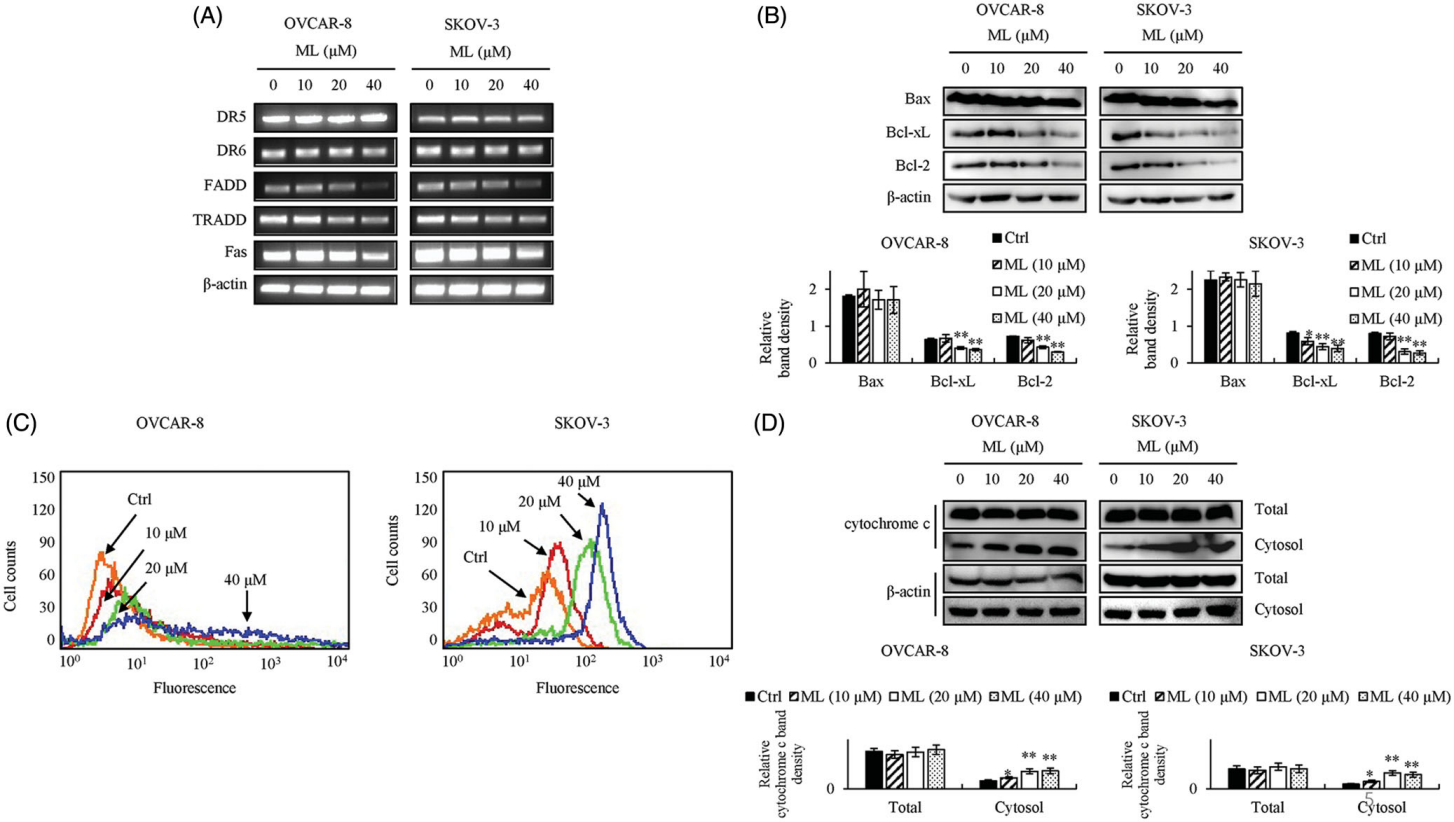
Effect of ML on apoptosis-related factors. (A) mRNA expression levels of factors related to extrinsic apoptosis analysed by RNA extraction and RT-PCR. (B) Western blotting of the intrinsic apoptotic factors was performed using whole cell lysates and specific antibodies. (C) Histogram profiles of JC-1 aggregation (FL-1, green), evaluated by flow cytometry. JC-1 aggregation indicates healthy cells, whereas JC-1 monomers indicate apoptotic cells. (D) Effect of ML on cytochrome c release. Cytosolic lysates were subjected to western blotting, as described in the ‘Materials and Methods’ section. Results represent the mean ± SEM of three experiments (significant *vs*. control, *p< 0.05, **p< 0.01).

Western blotting was performed to assess the expression of apoptosis-related factors. We investigated whether a caspase inhibitor, Z-VAD-FMK, would suppress the ML-induced intrinsic apoptotic pathway in both cell lines. Z-VAD-FMK effectively inhibited ML-induced cleavage of the caspases and PARP (Figure 3). Doxorubicin, a widely used antitumor agent, served as the positive control. PARP was cleaved after ML treatment, in a manner similar to that with doxorubicin (Figure 4). Considering the extrinsic apoptotic pathway, RT-PCR was performed to assess whether ML could induce the mRNA expression of extracellular ligands and receptors. ML could not induce the expression of death ligands or their receptors – DR5, DR6, Fas, TRADD and FADD (Figure 5(A)). Western blot analysis was performed to determine whether the ML-induced apoptosis was associated with the Bcl-2 family. Bax levels were unaltered; however, Bcl-2 and Bcl-xL were downregulated following ML treatment (Figure 5(B)). The release of cytochrome c into the cytosol was upregulated by ML (Figure 5(D)). This was verified *via* the assessment of cytochrome c levels in the cytosolic fractions of OVCAR-8 and SKOV-3 cells by western blot. Flow cytometric analyses revealed that 24 h ML treatment clearly induced loss of MMP in a dose-dependent manner, compared to control cells (Figure 5).

### ML induces G_2_/M phase arrest

**Figure 6.**
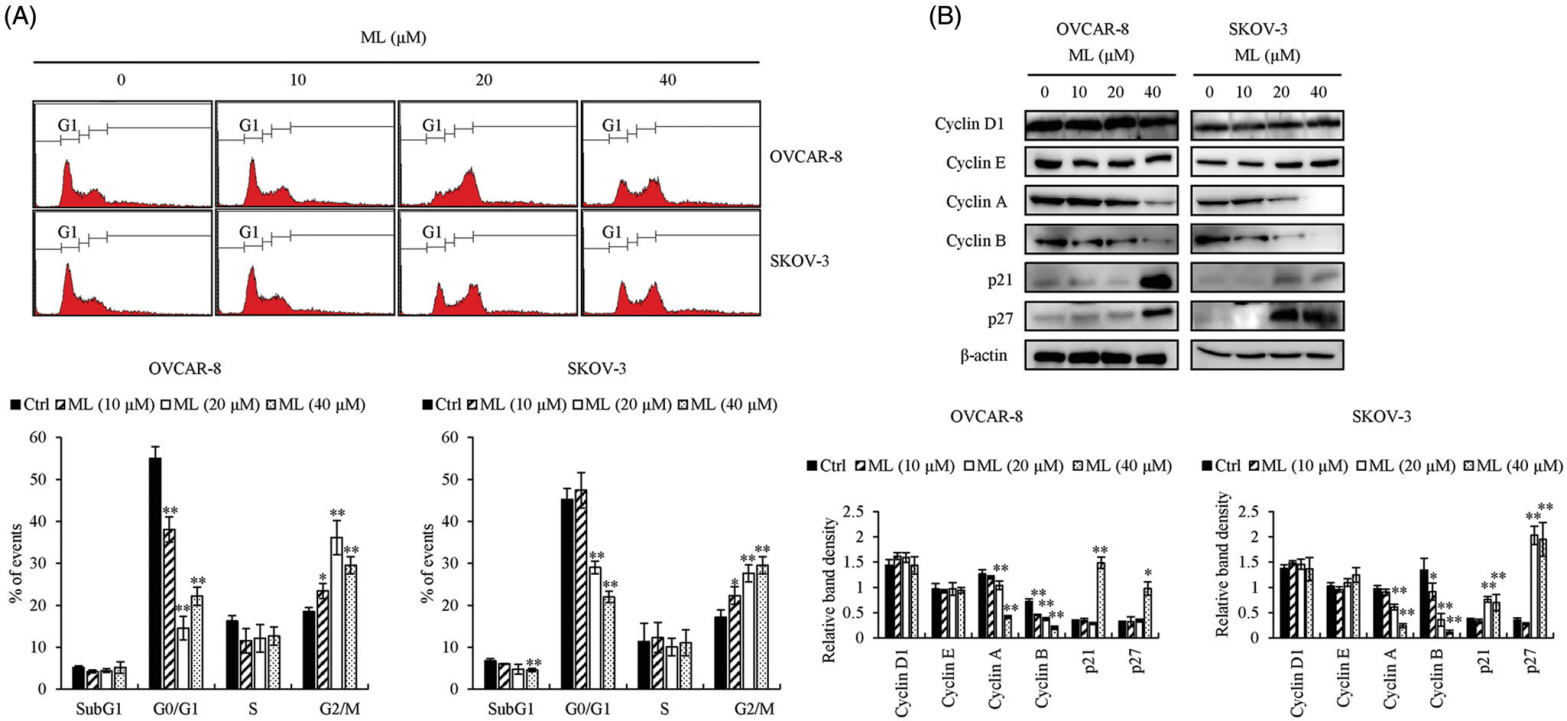
Effects of ML on cell cycle progression. OVCAR-8 and SKOV-3 cells were treated with the indicated amounts of ML for 24 h. (A) Cell cycle profiles using PI staining and flow cytometry. (B) Western blotting using whole cell lysates and specific antibodies for cell cycle regulatory factors cyclin D1, cyclin E, cyclin A, cyclin B, p27, p21 and β-actin. Results represent the mean ± SEM of three experiments (significant *vs*. control, *p< 0.05, **p< 0.01).

To address the mechanism responsible for ML-regulated cell cycle progression, OVCAR-8 and SKOV-3 cells were stained with PI and analysed by flow cytometry. As shown in Figure 6(A), ML-treated cells were significantly arrested at the G_2_/M phase, compared to the untreated group. Therefore, to investigate the mechanism by which ML caused G_2_/M phase arrest, the expression of cyclin D1, cyclin B, cyclin E and cyclin A were detected by immunoblotting. Interestingly, ML reduced cyclin A and B levels in a dose-dependent manner (Figure 6(B)). As expected, ML treatment upregulated the expression of cyclin-dependent kinase inhibitors p21 and p27 (Figure 6(B)).

### ML regulates on PI3K/Akt pathway

**Figure 7.**
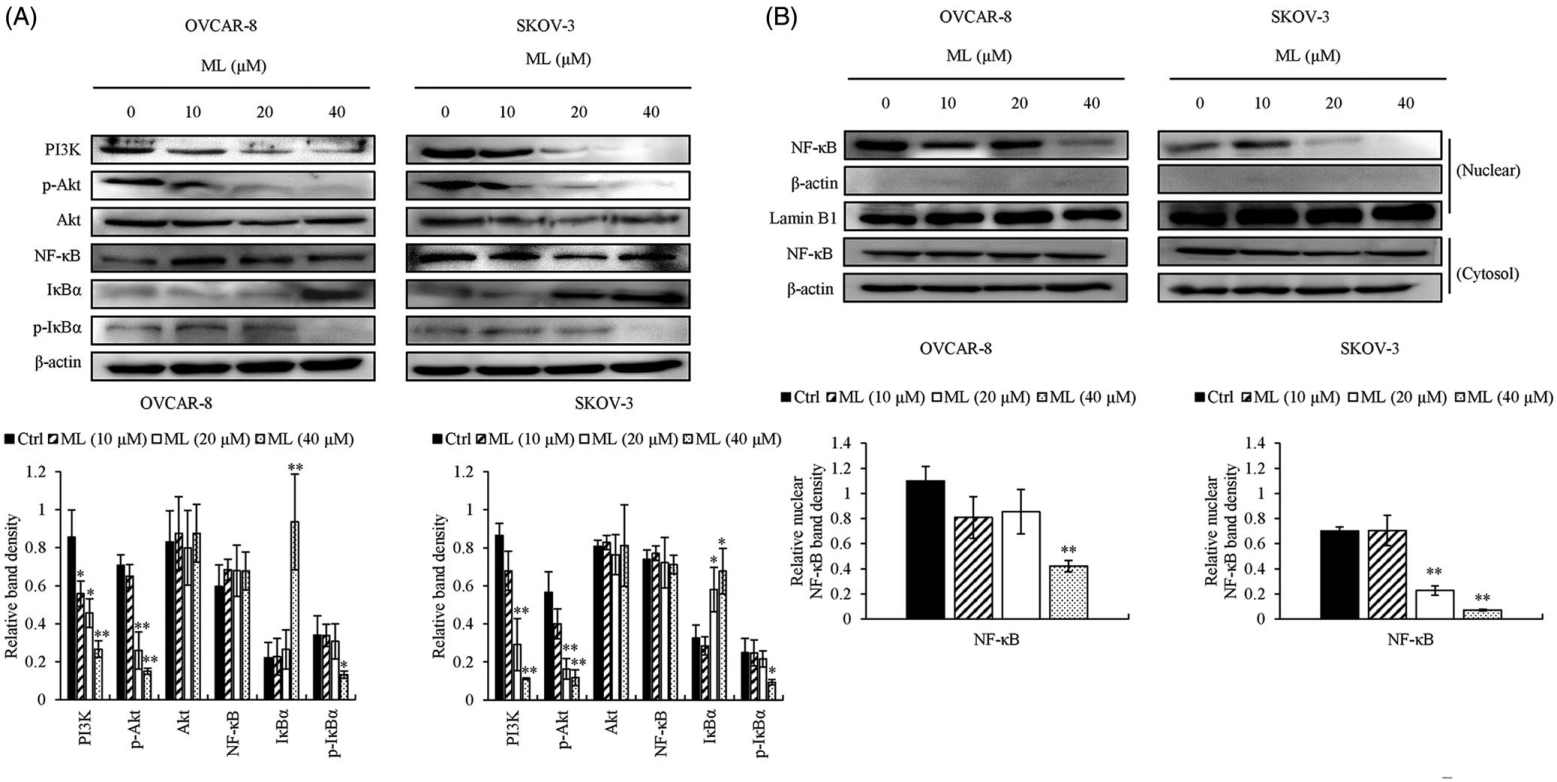
Effects of ML on the PI3K/Akt/NF-κB signalling pathway. OVCAR-8 and SKOV-3 cells were pre-treated with the indicated amounts of ML for 24 h. (A) Western blot analysis of PI3K/Akt/NF-κB, respectively, using whole-cell lysates and specific antibodies for PI3K, Akt, p-Akt, NF-κB, IκBα and p-IκBα. (B) Western blotting of the nuclear fraction of NF-κB. Lamin B1 and β-actin were used as loading controls. Results represent the mean ± SEM of three experiments (significant *vs*. control, *p< 0.05, **p< 0.01).

The PI3K/Akt signalling pathway plays a key role in cell survival, apoptosis, proliferation and tumour growth. To confirm whether treatment with ML affected the PI3K/Akt signalling pathway, we assessed PI3K and p-Akt levels; we observed that they were significantly downregulated in a dose-dependent manner, whereas the total level of Akt remained unaltered (Figure 7(A)).

### ML inhibits the NF-κB pathway

The NF-κB pathway is related to cell growth, survival and apoptosis and antiapoptosis. We performed western blotting on the cytoplasmic and nuclear fractions of the cells to examine the effects of ML on the NF-κB pathway. The translocation of NF-κB from the cytoplasm to the nucleus and the total level of p-IκBα were decreased after ML treatment, whereas the total level of IκBα was increased and NF-κB was unaltered (Figure 7(B)).

## Discussion

ML has been reported to modulate apoptosis *via* inhibition of STAT3 and MEG2 in a prostate cancer cell line and exert antiinflammatory effects, reducing croton oil-induced ear oedema in mice (Wang et al. 2008; Jin et al. 2018). However, the mechanism underlying ML-induced apoptosis has not been described in ovarian cancer cells; therefore, we investigated this phenomenon in detail.

Here, it was revealed that ML exerted anticancer effects by inducing apoptosis and G_2_/M phase arrest in human ovarian cancer cells through the PI3K/Akt pathway. The antiproliferative effect in ML-treated OVCAR-8 and SKOV-3 cells was evaluated at various time points. After 24 h, ML reduced cell proliferation (IC_50_=54.716 µM for OVCAR-8 and 60.708 µM for SKOV-3). These data mean that ML has antiproliferative effect on ovarian cancer cells in a dose- and time-dependent manner.

Apoptosis, a process of programmed cell death, is activated by two major pathways: the intrinsic and the extrinsic pathways (de Bruin and Medema 2008). It exhibits various characteristics, including cell shrinkage and externalization of phosphatidylserine (PS) that refers to the phenomenon of the PS inside the cell being translocated outside the cell membrane (Kerr et al. 1972; Sankari et al. 2012). This study found the proportion of apoptotic cells increased significantly after exposure to ML. Compared to control cells, the viability of treated cells was decreased; morphological changes between control and treated cells were also detected. Taken together, the results demonstrated that ML could reduce cell proliferation and induced apoptosis in ovarian cancer cells.

The extrinsic apoptotic pathway involves the death-inducing signalling complex (DISC), composed of the FADD, death receptor and caspase-8. DISC activates the caspase cascade, leading to apoptosis (de Bruin and Medema 2008). In the intrinsic apoptotic pathway, intracellular stimuli lead to mitochondrial dysfunction, resulting in the release of cytochrome c into the cytosol by an imbalance of pro-(Bax and Bak) or anti-apoptotic (Bcl-2 and Bcl-xL) Bcl-2 family proteins (Wei et al. 2001; Petros et al. 2004). Cytochrome c release was shown to be promoted by the binding of Bax to the mitochondria membrane; however, Bcl-2 inhibited cytochrome c efflux, forming a heterodimer with Bax and leading to the offset of pro-apoptotic effects (Huang et al. 2006). The released cytochrome c, through the dissipation of MMP, agglomerates with apoptotic protease-activating factor (Apaf-1) and caspase-9 and activates caspase-3, -6 and -7, which, in turn, cause PARP cleavage, leading to apoptosis (Li et al. 1997). After ML treatment, the ratio of Bax/Bcl-2 was increased and the MMP decreased as evidenced by the increased degree of PI staining and influx of cytochrome c into the cytosol. Moreover, ML-induced the activation of caspase-3 and -9 and PARP cleavage; this was inhibited by treatment with Z-VAD-FMK, an inhibitor of caspase-3 and -9. The level of extrinsic factors was unchanged. The intrinsic apoptotic effect was validated by the decrease in MMP and the downregulation of Bcl-2 protein expression after ML treatment. Altogether, our results indicate that ML-induced apoptosis is mediated by caspase-3 and -9; it can be suggested that this phenomenon is dependent on the intrinsic but not the extrinsic pathway.

Excessive cell cycle progression is a hallmark of cancer; its suppression results in the inhibition of tumour growth (Hanahan and Weinberg 2011). G_2_/M phase arrest can induce apoptosis and suppress proliferation (Pu et al. 2002; Chao et al. 2004). Each phase of the cell cycle is activated by a specific cyclin and can be regulated by cyclin-dependent kinase inhibitors p27 and p21. The cyclin B-CDK1 and cyclin A-CDK1 complexes facilitate the progression of the G_2_/M phase (Pietenpol and Stewart 2002). ML treatment-induced arrest of cells at G_2_/M phase, a potential target for inhibiting tumour growth. Furthermore, ML was found to upregulate p21 and p27 while downregulating the expression of cyclin A and B and inducing G_2_/M phase arrest, as reported in lung cancer cells (Shin et al. 2013). Overall, ML induced apoptosis *via* the intrinsic pathway and G_2_/M phase arrest. Therefore, we further investigated a key mechanism associated with apoptosis and G_2_/M phase arrest.

The PI3K/Akt pathway plays an important role in tumour survival and formation (Vivanco and Sawyers 2002). Particularly, it is involved in antiapoptotic activities, including NF-κB pathway activation, which regulates the expression of the Bcl-2 family and cell cycle regulators p21 and p27. IκB kinase (IKK) is also modulated by the PI3K/Akt pathway, resulting in translocation of NF-κB to the nucleus (Abukhdeir and Park 2008; Garcia et al. 2009). Inactive NF-κB (p50/p65 dimer) remains in the cytoplasm by binding to IκB. NF-κB, without bound IκB, is translocated into the nucleus where it activates several apoptotic or anti-apoptotic genes as a dual modulator (Kaltschmidt et al. 2000; Hamacher et al. 2008). The NF-κB pathway regulates intrinsic apoptotic factors, such as Bcl-2 and Bcl-xL (Khoshnan et al. 2000; Westerheide et al. 2001). Additionally, the cyclin-dependent kinase inhibitors p21 and p27 are phosphorylated by the Akt pathway and lose their ability to induce cell cycle arrest (Blagosklonny 2002). This study found that ML downregulated the expression of PI3K and p-Akt and reduced the nuclear NF-κB level, while the total NF-κB expression was maintained. Furthermore, the p-IκBα level was decreased, and IκBα was upregulated. These results demonstrated that ML blocks the PI3K/Akt pathway and NF-kB nuclear translocation. This might be involved in the downregulation of Bcl-2 family (Bcl-2/Bcl-xL) expression, and the CDK inhibitor (p21/p27) phosphorylation. Thus, it appears that ML induces apoptosis and G_2_/M phase arrest through the downregulation of the PI3K/Akt pathway.

## Conclusions

**Figure 8.**
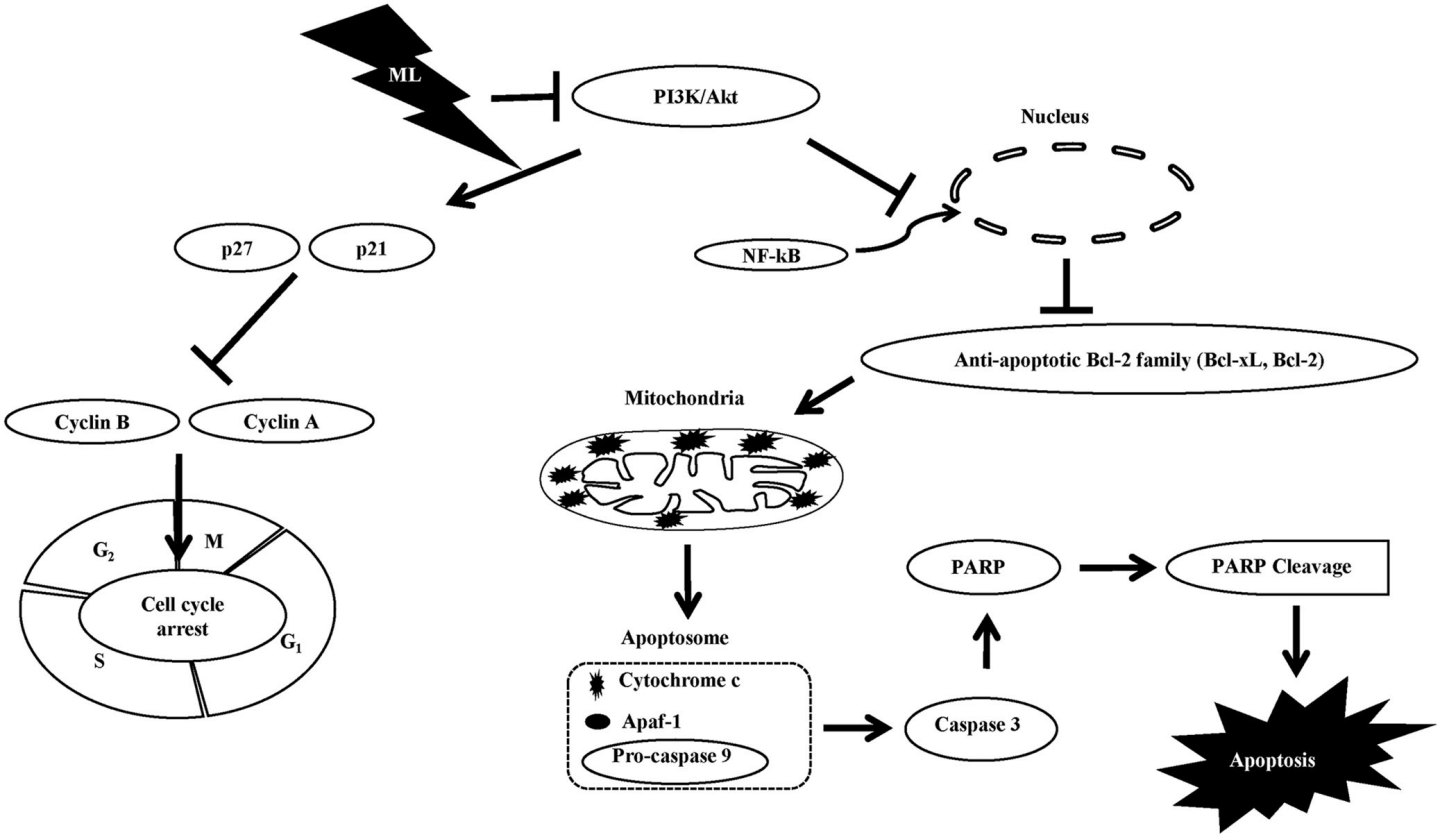
Schematic diagram of the potential ML-induced apoptotic pathway in ovarian cancer cells. ML inhibits the PI3K/Akt pathway and nuclear translocation of NF-κB, activates p21 and p27, and induces downregulation of the antiapoptotic factors Bcl-2 and Bcl-xL. The collapse of the MMP triggers an influx of cytochrome c into cytosol and induces activation of caspase-3 and -9 and PARP. Thus, ML induces cell cycle arrest and apoptosis *via* repression of the PI3K/Akt pathway and activation of the intrinsic apoptotic pathway *via* an imbalance in the members of the Bcl-2 family. Arrows indicate activation of the ML-induced signalling pathways; the closed lines indicate suppression by the ML-induced signal.

The study proved that ML induced apoptosis and G_2_/M phase arrest in ovarian cancer cells *via* inhibition of the PI3K/Akt pathway (Figure 8). This is the first study to demonstrate the molecular mechanism underlying the ML-mediated suppression of ovarian cancer cell viability; ML may, therefore, serve as a promising therapeutic agent for ovarian cancer.
